# Supplementary material for: Polymorphism in Toll-Like Receptors and Helicobacter Pylori Motility in Autoimmune Atrophic Gastritis and Gastric Cancer
Source: Cancers (Basel). 2019 May 10;11(5):648. doi: 10.3390/cancers11050648 (PMC6562993; doi:10.3390/cancers11050648)
Supplement: Supplementary file 1 [file cancers-11-00648-s001.pdf]

Supplementary Material

Polymorphism in Toll-Like Receptors and *Helicobacter Pylori* Motility in Autoimmune Atrophic Gastritis and Gastric Cancer

Valli De Re, Ombretta Repetto, Mariangela De Zorzi, Mariateresa Casarotto, Massimo Tedeschi, Paolo Giuffrida Marco Vincenzo Lenti, Raffaella Magris, Gianmaria Miolo, Cinzia Mazzon, Giorgio Zanette, Lara Alessandrini, Vincenzo Canzonieri, Laura Caggiari, Stefania Zanussi, Agostino Steffan, Antonio Di Sabatino and Renato Cannizzaro

Table S1. Genotype and allele frequencies of the TLR gene polymorphisms in AAG compared to (A) HD and to (B) GC.

		**A**						
		**AAG (*n* = 67)**		**HD (*n* = 97)**				
	**Genotype**	**count**	**frequency**	**count**	**frequency**	***p***	***Corrected p-value***	***Age-and sex-adjusted OR (95% CI) ****
**TLR1** rs4833095	T/T	27	0.40	33	0.34			*1.00*
	C/T	29	0.43	47	0.48	0.34	*0.51*	*2.64 (0.43-16.32)*
	C/C	11	0.16	17	0.18			*1.21 (0.10-14.12)*
	T	83	0.62	113	0.58	0.2		
	C	51	0.38	81	0.42			
**TLR2** rs3804099	T/T	17	0.25	28	0.29			*1.00*
	C/T	37	0.55	54	0.56	0.77	*0.62*	*0.90 (0.12-6.62)*
	C/C	13	0.19	15	0.15			*0.26 (0.01-6.54)*
	T	71	0.53	181	0.55	0.74		
	C	63	0.47	147	0.45			
**TLR2** 196-174del	A/A	49	0.73	68	0.70			*1.00*
	T/A	17	0.25	28	0.29	0.86	*0.64*	*2.35 (0.38-14.5)*
	T/T	1	0.01	1	0.01			--
	A	115	0.86	279	0.85	0.83		
	T	19	0.14	49	0.15			
**TLR4** rs4986790	A/A	60	0.9	85	0.88	0.7	*0.33*	*1.00*
	A/G	7	0.1	12	0.12			*4.44 (0.48-41.0)*
	A	127	0.95	182	0.94	0.71		
	G	7	0.05	12	0.06			
**TLR4** rs4986791	C/C	60	0.9	85	0.88	0.7	*0.33*	*1.00*
	C/T	7	0.1	12	0.12			*4.44 (0.48-41.0)*
	C	127	0.95	182	0.94	0.71		

	T	7	0.05	12	0.06			
**TLR5** rs5744174	T/T	23	0.34	39	0.40			*1.00*
	C/T	37	0.55	38	0.40	**0.04**	***0.06***	***1.57 (1.0-3.09)***
	C/C	7	0.1	20	0.20			*0.57 (0.21-1.54)*
	T	83	0.62	116	0.60	0.69		
	C	51	0.38	78	0.40			
	Overdominant model #							
	T/T + C/C	30	0.45	59	0.61			*1.00*
	C/T	37	0.55	38	0.39	**0.04**	***0.021***	***1.85 (1.00-3.44)***
**TLR8°** rs3764880	***Female***							
	A/A	30	0.58	22	0.53			*1.00*
	A/G	18	0.35	13	0.30	0.32	*0.35*	*1.99 (0.66-6.02)*
	G/G	4	0.08	7	0.16			*2.66 (0.48-14.6)*
	A	78	0.75	57	0.68			
	G	26	0.25	27	0.32	0.28		
	Male							
	A	11	0.73	41	0.75	0.89	*0.10*	*1.00*
	G	4	0.27	14	0.25			*0.94 (0.38-2.34)*
**TLR9** rs5743836	T/T	55	0.82	70	0.72			*1.00*
	T/C	12	0.18	25	0.26	0.16	*0.69*	*2.06 (0.40-10.60)*
	C/C	0	0	2	0.02			--
	T	122	0.91	165	0.88	0.11		
	C	12	0.09	29	0.12			

B

		AAG (n = 67)		GC (n = 114)				
	**Genotype**	**count**	**frequency**	**count**	**frequency**	***p***	***Corrected p-value***	***Age-and sex-adjusted OR (95% CI) ****
**TLR1** rs4833095	T/T	28	0.42	34	0.30			*1.00*
	C/T	30	0.44	65	0.57	0.44	*0.32*	*2.02 (0.79-5.14)*
	C/C	9	0.14	15	0.13			*1.30 (0.34-4.94)*
	T	86	0.64	133	0.58	0.27		
	C	48	0.36	95	0.42			
**TLR2** rs3804099	T/T	17	0.25	32	0.28			*1.00*
	C/T	37	0.55	62	0.54	0.91	*0.82*	*0.89 (0.43-1.82)*
	C/C	13	0.19	20	0.18			*0.82 (0.32-2.03)*
	T	71	0.53	126	0.55	0.67		
	C	63	0.47	102	0.45			
**TLR2** 196 -174del	A/A	49	0.73	78	0.68			*1.00*
	T/A	17	0.25	31	0.27	0.50	*0.79*	*1.14 (0.28-4.61)*

	T/T	1	0.01	5	0.05			*NA*
	A	115	0.86	187	0.82	0.35		
	T	19	0.14	41	0.18			
	A/A	60	0.9	102	0.89	0.99	*0.34*	*1.00*
**TLR4**	A/G	7	0.1	12	0.11			*3.19 (0.27-37.15)*
rs4986790	A	127	0.95	216	0.95	0.99		
	G	7	0.05	12	0.05			
	C/C	60	0.9	102	0.89	0.99	*0.34*	*1.00*
**TLR4**	C/T	7	0.1	12	0.11			*3.19 (0.27-37.15)*
rs4986791	C	127	0.95	216	0.95	0.99		
	T	7	0.05	12	0.05			
	T/T	23	0.34	47	0.41			*1.00*
	C/T	37	0.55	44	0.39	**0.07**	***0.06***	*0.68 (0.33-1.41)*
	C/C	7	0.1	23	0.2			*2.26 (0.77-6.66)*
**TLR5**	T	83	0.62	138	0.61	0.22		
rs5744174	C	51	0.38	111	0.47			
	Recessive# model							
	T/T + C/T	60	0.90	91	0.80	**0.07**	***0.03***	*1.00*
	C/C	7	0.10	23	0.20			***2.81 (1.04-7.62)***
	Female							
	A/A	30	0.58	19	0.44	0.37	*0.37*	*1.00*
	A/G	18	0.35	21	0.49			*1.85 (0.78-4.34)*
	G/G	4	0.08	3	0.08			*1.19 (0.24-5.95)*
**TLR8°**	A	78	0.75	59	0.68	0.22		
rs3764880	G	26	0.25	27	0.32			
	Male							
	A	11	0.73	50	0.70	0.73	*0.77*	*1.00*
	G	4	0.27	21	0.30			*1.20 (0.34-4.27)*
	T/T	*55*	*0.82*	*80*	*0.70*			*1.00*
	T/C	*12*	*0.18*	*30*	*0.26*	*0.28*	*0.31*	*2.42 (0.62-9.45)*
TLR9	*C/C*	*0*	*0*	*4*	*0.04*			*--*
rs5743836	*T*	*122*	*0.91*	*190*	*0.83*	***0.04***		
	C	*12*	*0.09*	*38*	*0.17*			

* Analysis of association are performed by regression analysis; Corrected *p*-value for age and sex. # five inheritance models are fitted which correspond to different grouping of the genotypes: codominant, dominant, recessive, co-dominant and additive. Table reports the best model for a specific polymorphism based on akaike's information criterion (AIC). ° TLR8 stratified by gender.

Table S2. List of flagellins (A and/or B) identified in *Helicobacter pylori* isolates.

Gel portion nr.[a)]	Database	Accession	Mass	Protein annotation	Score	Matches	Seq.[b)]	Seq. cov. %[c)]
1	No detection.							
2	SwissProt	2::FLAA_HELPJ	53252	**Flagellin A** OS = Helicobacter pylori (strain J99 / ATCC 700824)	303	13	10	17
3	SwissProt	2::FLAA_HELPJ	53252	**Flagellin A** OS = Helicobacter pylori (strain J99 / ATCC 700824)	143	13	8	14
	SwissProt	2::FLAB_HELPJ	53937	**Flagellin B** OS = Helicobacter pylori (strain J99 / ATCC 700824)	88	9	7	15
4	NCBInr	1::gi|261839570	53253	**Flagellin A** [Helicobacter pylori 52]	1086	60	18	34
	NCBInr	1::gi|317180372	53266	**Flagellin A** [Helicobacter pylori F32]	1059	55	19	39
	NCBInr	1::gi|188527549	53278	**Flagellin A** [Helicobacter pylori Shi470]	781	48	17	29
	NCBInr	1::gi|108562540	53951	**Fllagellin B** [Helicobacter pylori HPAG1]	540	39	17	33
5	SwissProt	2::FLAB_HELPJ	53937	**Flagellin A** OS = Helicobacter pylori (strain J99 / ATCC 700824)	272	14	8	30
6	SwissProt	2::FLAB_HELPJ	53937	**Flagellin A** OS = Helicobacter pylori (strain J99 / ATCC 700824)	549	28	16	30
	NCBInr	1::gi|261839570	53253	**Flagellin A** [Helicobacter pylori 52]	508	30	16	30

[a)] gel portion nr., gel portion numbers refer to Figure 3; [b)] Seq., sequences; [c)] Seq. cov. %, percentage of sequence coverage.

Table 3. Primers used in the amplification and sequencing PCR of *H. pylori flaA* gene.

Primer	Coordinates†	Primer sequences (5′→3′)	Application
FlaA F	597956-597981	TGGCTTTTCAGGTCAATACAAATATC	Amplification and cycle sequencing
FlaA R	599451-599476	GCCTTAAGATATTTGRTGAACGGTA *	Amplification and cycle sequencing
FlaA 451 F	598437-598456	CGGCTCTACCACTTCCGATA	Cycle sequencing
FlaA 716 F	598650-598669	TTATGCGAGCGTTATCACCA	Cycle sequencing
FlaA 1226 F	599160-599179	GATCGCTAGCGGTAACCAAA	Cycle sequencing
FlaA 289 R	598256-598275	TTCCGTAGTTTGCCCATCTT	Cycle sequencing
FlaA 551 R	598466-598485	GCCTGTAGCGATACGAACCT	Cycle sequencing
FlaA 802 R	598769-598788	GATCGCTGCGACTAACCTTC	Cycle sequencing

†, coordinates are referred to *H. pylori J99* strain genome; *, R = A or G, degenerate base.

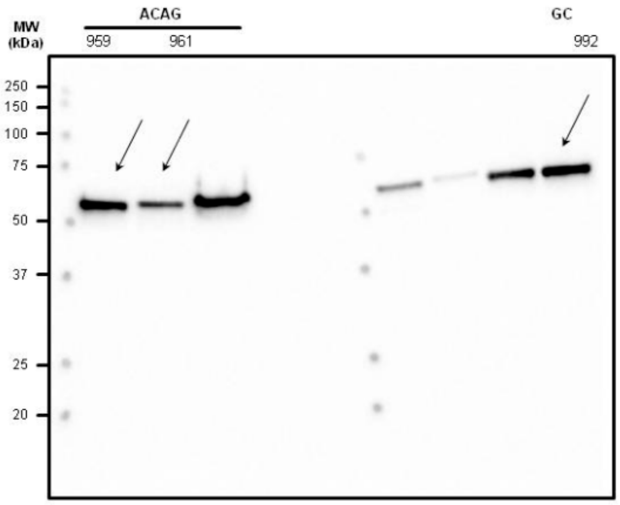

Figure S1. Proteomic characterization of flagellin A. The whole immunoblotting from which figure 2C comes is shown, and arrows indicate signal of proteins cross-reacting with the anti-flagellin antibody at around 54 kDa in *H. pylori* isolated from either AAG (samples 959 and 961) and on GC (sample 992).

© 2019 by the authors. Licensee MDPI, Basel, Switzerland. This article is an open access article distributed under the terms and conditions of the Creative Commons Attribution (CC BY) license (http://creativecommons.org/licenses/by/4.0/).
